# Advances in molecular-based personalized non-small-cell lung cancer therapy: targeting epidermal growth factor receptor and mechanisms of resistance

**DOI:** 10.1002/cam4.506

**Published:** 2015-08-26

**Authors:** Robert M Jotte, David R Spigel

**Affiliations:** 1 Rocky Mountain Cancer CentersLone Tree, Colorado; 2 Sarah Cannon Research InstituteNashville, Tennessee

**Keywords:** Afatinib, dacomitinib, erlotinib, gefitinib, non-small-cell lung cancer, resistance

## Abstract

Molecularly targeted therapies, directed against the features of a given tumor, have allowed for a personalized approach to the treatment of advanced non-small-cell lung cancer (NSCLC). The reversible epidermal growth factor receptor (EGFR) tyrosine kinase inhibitors (TKIs) gefitinib and erlotinib had undergone turbulent clinical development until it was discovered that these agents have preferential activity in patients with NSCLC harboring activating *EGFR* mutations. Since then, a number of phase 3 clinical trials have collectively shown that EGFR-TKI monotherapy is more effective than combination chemotherapy as first-line therapy for *EGFR* mutation-positive advanced NSCLC. The next generation of EGFR-directed agents for *EGFR* mutation-positive advanced NSCLC is irreversible TKIs against EGFR and other ErbB family members, including afatinib, which was recently approved, and dacomitinib, which is currently being tested in phase 3 trials. As research efforts continue to explore the various proposed mechanisms of acquired resistance to EGFR-TKI therapy, agents that target signaling pathways downstream of EGFR are being studied in combination with EGFR TKIs in molecularly selected advanced NSCLC. Overall, the results of numerous ongoing phase 3 trials involving the EGFR TKIs will be instrumental in determining whether further gains in personalized therapy for advanced NSCLC are attainable with newer agents and combinations. This article reviews key clinical trial data for personalized NSCLC therapy with agents that target the EGFR and related pathways, specifically based on molecular characteristics of individual tumors, and mechanisms of resistance.

## Introduction

At least 85% of lung cancers are histologically classified as non-small-cell lung cancer (NSCLC), often requiring systemic therapy for advanced disease 1. As conventional chemotherapy with platinum-based doublets is associated with improved clinical outcomes, but also potentially higher toxicity 1, the evaluation of molecularly targeted therapies has led to extensive investigation and several U.S. Food and Drug Administration (FDA) approvals for advanced NSCLC 2–5.

This review article describes the framework upon which epidermal growth factor receptor tyrosine kinase inhibitor (EGFR-TKI) therapy has been built and summarizes the ongoing work in the development of personalized medicine in the EGFR-TKI field.

## EGFR and ErbB Family Pathways

### Overview

**Figure 1:**
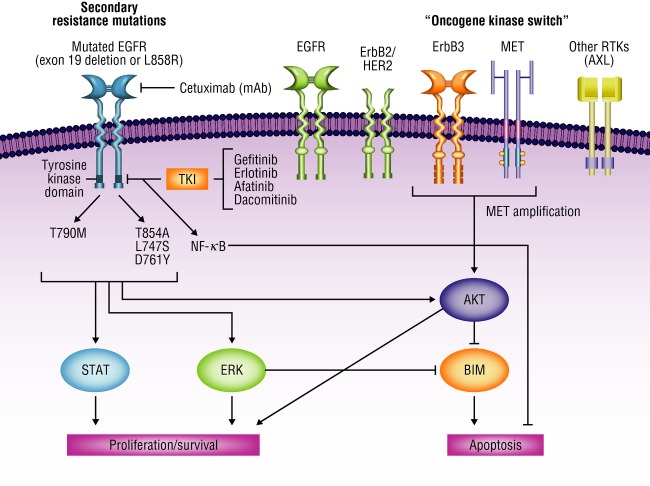
Mechanisms of acquired resistance to gefitinib/erlotinib in *EGFR*-mutated NSCLC. EGFR, epidermal growth factor receptor; ErbB3, v-erb-b2 avian erythroblastic leukemia viral oncogene homolog 3; NSCLC, non-small-cell lung cancer; RTK, receptor tyrosine kinase; MET, met proto-oncogene; AXL, AXL receptor tyrosine kinase; mAb, monoclonal antibody; TKI, tyrosine kinase inhibitor; NF-*κ*B, nuclear factor kappa-light-chain-enhancer of activated B cells; AKT, v-akt murine thymoma viral oncogene homolog 1; STAT, signal transducers and activation of transcription; ERK, extracellular signal-regulated kinase; BIM, BCL2-like 11 (apoptosis facilitator). Adapted from Nguyen et al. 43, with permission from Elsevier.

EGFR, or human epidermal growth factor receptor 1 (HER1)/ErbB1, is the first of the four receptor tyrosine kinases (TKs) within the ErbB family 6. Autophosphorylation of EGFR and other family members is a key step toward activation of several pathways involved in cellular proliferation, including the retrovirus-associated DNA sequences (Ras)/v-raf 1 murine leukemia viral oncogene homolog 1 (Raf)/mitogen-activated protein kinase (MAPK) pathway and phosphoinositide-3 kinase (PI3K)/protein kinase B (Akt) pathway 7. Additional downstream effects of ErbB family signaling include signal transducers and activation of transcription (STAT) recruitment and phosphorylation (Fig.1) 7,8.

### Role of EGFR and the ErbB family in NSCLC therapy

Increased EGFR expression and its association with advanced disease in NSCLC 9 prompted early efforts toward clinical development of agents targeting the EGFR pathway. The first-generation reversible EGFR TKIs gefitinib (Iressa®, AstraZeneca, Wilmington, DE) 2 and erlotinib (Tarceva®, Genentech, South San Francisco, CA) 4 were the first EGFR-directed therapies to be approved by the FDA in NSCLC. Of note, gefitinib use has been discontinued in the United States. Based on data establishing that EGFR TKIs confer the most benefit when used in molecularly selected patients, erlotinib was granted an additional indication by the FDA in May 2013 for first-line treatment of patients with metastatic NSCLC whose tumors have *EGFR* exon 19 deletions or exon 21 (L858R) mutations as detected by an FDA-approved test 4. In July 2013, the irreversible ErbB family TKI afatinib (Gilotrif®, Boehringer Ingelheim, Ingelheim, Germany) was approved by the FDA in the same setting and also accompanied by an FDA-approved mutational test 10.

### Discovery and implications of activating EGFR mutations

A key discovery toward personalized therapy for NSCLC was the association between activating somatic *EGFR* mutations and response to gefitinib and erlotinib 11, observed at a higher rate in Asian compared with Western populations 12. Known *EGFR*-activating mutations are located on exons 18–21 within the TK domain 13, with ∼90% estimated to involve deletions in exon 19 and point mutations in exon 21 12. This molecular characterization served to explain the clustering of responses in early clinical trials among patients with certain characteristics, including East Asian ethnicity, adenocarcinoma histology, female gender, and nonsmoking history 13,14. While *EGFR* mutations are more commonly observed in patients with these clinical characteristics (i.e., Asian ethnicity, adenocarcinoma histology, etc.), they can occur in patients who do not fit these characteristics as well. In related findings, it is now known that *KRAS* mutations are often mutually exclusive with *EGFR*-activating mutations and may be associated with EGFR-TKI resistance 1,15.

The criticality of *EGFR* mutations in predicting response to EGFR TKIs makes molecular testing important in both clinical trials and clinical practice 1. Per the 2015 National Comprehensive Cancer Network (NCCN) guidelines 1, routine *EGFR* mutation testing is recommended in NSCLC of adenocarcinoma, large cell, or unknown histology, but not in squamous cell carcinoma (except in never smokers and mixed histology or small biopsy specimens) given its rarity in this subtype. The 2013 guidelines from the College of American Pathologists, International Association for the Study of Lung Cancer, and Association for Molecular Pathology recommend *EGFR* mutation testing for adenocarcinomas and mixed lung cancers with an adenocarcinoma component regardless of clinical characteristics or risk factors 16. Guidelines recommend laboratories use validated molecular testing methods with sufficient performance characteristics 16; options include direct sequencing 17, immunohistochemistry (IHC) 17, and polymerase chain reaction-based analysis (e.g., Scorpion Amplification Refractory Mutation System technology [DxS]) 18. From a clinical practice standpoint, *EGFR* reflex testing of resected pulmonary adenocarcinoma has demonstrated feasibility 19.

### Phase 3 clinical trials in molecularly selected NSCLC populations

Available data from completed phase 3 trials of EGFR or ErbB family TKIs in *EGFR* mutation-positive NSCLC or clinically selected populations are summarized in Tables1 and 2 and discussed below, along with recent phase 2 data for the newer generation of irreversible agents.

**Table 1:** Phase 3 clinical trial results for EGFR or ErbB family TKIs as first-line therapy in molecularly selected NSCLC

Trial	Study	Treatment arms	RR, %	Median PFS	Median OS	Most common grade ≥3 AEs (TKI vs. chemotherapy)
Gefitinib						
WJTOG3405 22,23,71	Japanese study of 177 patients with *EGFR* mutations	Gefitinib vs. cisplatin/docetaxel	62.1 vs. 32.2 (*P *<* *0.0001)	9.2 vs. 6.3 months (HR, 0.489; 95% CI, 0.336–0.710; *P *<* *0.0001)	34.8 vs. 37.3 months (HR, 1.252; 95% CI, 0.883–1.775)	ALT elevation (24% vs. 2%), AST elevation (14% vs. 1%)
NEJ002 20,21	Japanese study of 230 patients with *EGFR* mutations	Gefitinib vs. carboplatin/paclitaxel	73.7 vs. 30.7 (*P *<* *0.001)	10.8 vs. 5.4 months (HR, 0.322; 95% CI, 0.236–0.438; *P *<* *0.001)	27.7 vs. 26.6 months (HR, 0.887; 95% CI, 0.634–1.241; *P *=* *0.483)	Aminotransferase elevation (26% vs. 1%), appetite loss (5% vs. 6%)
IPASS 24	East Asian study of 261 patients with *EGFR* mutations	Gefitinib vs. carboplatin/paclitaxel	71.2 vs. 47.3 (*P *<* *0.001)	HR, 0.48; 95% CI, 0.36–0.64; *P *<* *0.001)	HR, 0.91; 95% CI, 0.76–1.10	Diarrhea (3.8% vs. 1.4%), neutropenia (3.7% vs. 67.1%), rash/acne (3.1% vs. 0.8%)
First-SIGNAL 26	Korean study of 42 patients with *EGFR* mutations	Gefitinib vs. cisplatin/gemcitabine	84.6 vs. 37.5 (*P *=* *0.002)	8.0 vs. 6.3 months (HR, 0.544; 95% CI, 0.269–1.100; *P *=* *0.086)	27.2 vs. 25.6 months (HR, 1.043; 95% CI, 0.498–2.182)	Rash (29.3% vs. 2.0%), anorexia (13.8% vs. 57.3%), AST (11.3% vs. 2.0%)
Erlotinib						
OPTIMAL 27	Chinese study of 165 patients with *EGFR* mutations	Erlotinib vs. carboplatin/gemcitabine (up to four cycles)	83 vs. 36 (*P *<* *0.0001)	13.1 vs. 4.6 months (HR, 0.16; 95% CI, 0.10–0.26; *P *<* *0.0001)	NR	ALT elevation (4% vs. 1%), skin rash (2% vs. 0%)
EURTAC 28,1	European study of 173 patients with *EGFR* mutations	Erlotinib vs. platinum-based chemotherapy (up to four cycles)	58 vs. 15 (*P *<* *0.0001)	9.7 vs. 5.2 months (HR, 0.37; 95% CI, 0.25–0.54; *P *<* *0.0001)	19.3 vs. 19.5 months (HR, 1.04; 95% CI, 0.65–1.68; *P *=* *0.87)	Rash (13% vs. 0%), fatigue (6% vs. 20%)
Afatinib						
LUX-Lung 6 33,34,2	Asian study of 364 patients with *EGFR* mutations	Afatinib vs. cisplatin/gemcitabine (up to six cycles)	66.9 vs. 23.0 (*P *<* *0.0001)	11.0 vs. 5.6 months (HR, 0.28; 95% CI, 0.20–0.39; *P *<* *0.0001)	23.1 vs. 23.5 months (HR, 0.93; *P *=* *0.6137)	Rash/acne (14.6% vs. 0%), diarrhea (5.4% vs. 0%), stomatitis/mucositis (5.4% vs. 0%)
LUX-Lung 3 34,35,2	Global study of 345 patients with *EGFR* mutations	Afatinib vs. cisplatin/pemetrexed (up to six cycles)	56 vs. 23 (*P *=* *0.001)	11.1 vs. 6.9 months (HR, 0.58; 95% CI, 0.43–0.78; *P *=* *0.001)	28.2 vs. 28.2 months (HR, 0.88; *P *=* *0.3850)	Rash (16.2% vs. 0%), diarrhea (14.4% vs. 0%), paronychia (11.4% vs. 0%), stomatitis/mucositis (8.7% vs. 0.9%)

EGFR, epidermal growth factor receptor; TKIs, tyrosine kinase inhibitors; NSCLC, non-small-cell lung cancer; RR, response rate; PFS, progression-free survival; OS, overall survival; AEs, adverse events; HR, hazard ratio; CI, confidence interval; ALT, alanine aminotransferase; AST, aspartate aminotransferase; NR, not reported.

1 Investigator assessed.

2 Independent review.

**Table 2:** PFS and OS from phase 3 clinical trials for EGFR or ErbB family TKIs for NSCLC by Del19 and L858R *EGFR* mutation subtypes

Trial	Treatment arms	Median PFS, Del19	Median PFS, L858R	Median OS, Del19	Median OS, L858R
Gefitinib					
WJTOG3405 22	Gefitinib vs. cisplatin/docetaxel (up to six cycles)	9.0 vs. 6.0 months (HR, 0.45; 95% CI, 0.27–0.77)	9.6 vs. 6.7 months (HR, 0.51; 95% CI, 0.29–0.90)	NR	NR
NEJ002 20,21	Gefitinib vs. carboplatin/paclitaxel (up to six cycles)	11.5 months for gefitinib (HR, 0.35; 95% CI, 0.23–0.52)	10.8 months for gefitinib (HR, 0.32; 95% CI, 0.20–0.50)	NR	NR
IPASS 24,25,72	Gefitinib vs. carboplatin/paclitaxel (up to six cycles)	HR, 0.38; 95% CI, 0.26–0.56	HR, 0.55; 95% CI, 0.35–0.87	HR, 0.79; 95% CI, 0.54–1.15	HR, 1.44; 95% CI, 0.90–2.30
Erlotinib					
OPTIMAL 27	Erlotinib vs. carboplatin/gemcitabine (up to four cycles)	HR, 0.13; 95% CI, 0.07–0.25	HR, 0.26; 95% CI, 0.14–0.49	NR	NR
EURTAC 4,28	Erlotinib vs. platinum-based chemotherapy (up to four cycles)	11.0 vs. 4.6 months (HR, 0.30; 95% CI, 0.18–0.50; *P *<* *0.0001)	8.4 vs. 6.0 months (HR, 0.55; 95% CI, 0.29–1.02; *P *=* *0.0539)	HR, 0.94; 95% CI, 0.57–1.54	HR, 0.99; 95% CI, 0.56–1.76
Afatinib					
LUX-Lung 6 33,34	Afatinib vs. cisplatin/gemcitabine (up to six cycles)	HR, 0.20^1^; 95% CI, 0.13–0.33	HR, 0.32^1^; 95% CI, 0.19–0.52	31.4 vs. 18.4 months (HR, 0.64; 95% CI, 0.44–0.94; *P *=* *0.0229)	HR, 1.22; 95% CI, 0.81–1.83
LUX-Lung 3 34,35	Afatinib vs. cisplatin/pemetrexed (up to six cycles)	HR, 0.28^1^; 95% CI, 0.18–0.44	HR, 0.73^1^; 95% CI, 0.46–1.17	33.3 vs. 21.1 months (HR, 0.54; 95% CI, 0.36–0.79; *P *=* *0.0015)	HR, 1.30; 95% CI, 0.80–2.11

PFS, progression-free survival; OS, overall survival; EGFR, epidermal growth factor receptor; TKIs, tyrosine kinase inhibitors; NSCLC, non-small-cell lung cancer; HR, hazard ratio; CI, confidence interval; NR, not reported.

1 Independent review.

#### Reversible EGFR TKIs (gefitinib and erlotinib)

In the phase 3 NEJ002 trial of gefitinib versus carboplatin/paclitaxel in 230 patients with *EGFR*-activating mutations 20, gefitinib significantly improved median progression-free survival (PFS, 10.8 vs. 5.4 months; *P *<* *0.001) and response rate (RR, 73.7% vs. 30.7%; *P *<* *0.001). Based on updated overall survival (OS) results, median OS was 27.7 months with gefitinib and 26.6 months with chemotherapy (*P *=* *0.483), with this lack of difference potentially attributable to a high rate of crossover to gefitinib in the control arm 21. WJTOG3405 was a phase 3 trial of gefitinib versus cisplatin/docetaxel in 177 Japanese patients with advanced or recurrent NSCLC with *EGFR*-activating mutations 22. As in NEJ002, gefitinib significantly prolonged median PFS (9.2 vs. 6.3 months; *P *<* *0.0001) and increased RR (62.1% vs. 32.2%; *P *<* *0.0001) 22; median OS was 34.8 months with gefitinib versus 37.3 months with cisplatin/docetaxel 23.

Results of the IPASS phase 3 trial, conducted in a population of 1217 patients with clinical characteristics predictive of *EGFR* mutations (namely East Asian nonsmokers with adenocarcinoma), provide additional support for the activity of gefitinib in this setting 24,25. Gefitinib was associated with a median PFS of 5.7 months that was noninferior to carboplatin/paclitaxel (5.8 months; *P *<* *0.001 for noninferiority) and a significantly higher 12-month PFS (24.9% vs. 6.7%, respectively; *P *<* *0.001) and RR (43.0% vs. 32.2%; *P *<* *0.001) 24. Median OS was 18.8 months for gefitinib and 17.4 months for chemotherapy (*P *=* *0.109) 25. In the subgroup of patients with *EGFR*-activating mutations (*n* = 261), PFS was significantly longer with gefitinib versus chemotherapy (*P *<* *0.001) and the RR was significantly higher (71.2% vs. 47.3%; *P *<* *0.001); conversely, in the *EGFR* mutation-negative subgroup, PFS was significantly shorter (*P *<* *0.001) and the RR was significantly lower with gefitinib versus chemotherapy (1.1% vs. 23.5%; *P *=* *0.001) 24. OS was similar regardless of treatment in *EGFR* mutation-positive patients (21.6 vs. 21.9 months; *P *=* *0.990), but was likely confounded by a high rate of crossover to EGFR-TKI therapy 25. Exploration of biomarkers in IPASS found that gefitinib significantly prolonged PFS in cases where tumors had high *EGFR* gene copy number and an *EGFR* mutation, but not when high *EGFR* gene copy number was unaccompanied by an *EGFR* mutation; in the latter subset, PFS was significantly shorter with gefitinib versus carboplatin/paclitaxel.

The most recently published phase 3 data for first-line gefitinib versus chemotherapy for advanced NSCLC are from the Korean First-SIGNAL phase 3 trial, which included never smokers with lung adenocarcinoma 26. In the overall study population (*N* = 309), there were no significant benefits for gefitinib versus cisplatin/gemcitabine with respect to RR (55.4% vs. 46.0%; *P *=* *0.101), PFS (5.8 vs. 6.4 months; *P *=* *0.138), or the primary endpoint of OS (22.3 vs. 22.9 months; *P *=* *0.604). Among 42 patients with *EGFR* mutation-positive disease, the RR was significantly higher with gefitinib versus cisplatin/gemcitabine (84.6% vs. 37.5%; *P *=* *0.002), but PFS was not significantly different (*P *=* *0.086).

As with gefitinib, phase 3 data are available to support the activity of erlotinib versus doublet chemotherapy as first-line therapy for *EGFR* mutation-positive NSCLC. The OPTIMAL trial compared erlotinib against carboplatin/gemcitabine as first-line therapy in 165 Chinese patients with *EGFR*-activating mutations, with significant benefits for erlotinib with respect to PFS (13.1 vs. 4.6 months; *P *<* *0.0001) and RR (83% vs. 36%; *P *<* *0.0001) observed 27. Similarly, the EURTAC phase 3 trial assessed erlotinib versus first-line platinum-based chemotherapy in 174 patients with *EGFR* mutation-positive advanced NSCLC 28. At preplanned interim analysis, erlotinib significantly improved the investigator-assessed primary endpoint of PFS (9.4 vs. 5.2 months; *P *<* *0.0001), prompting early closure of the study. The final results of EURTAC (Table1) were consistent with those in the interim analysis.

Both gefitinib and erlotinib continue to be studied in *EGFR* mutation-positive advanced NSCLC in ongoing phase 3 and 4 trials (Table3).

**Table 3:** Ongoing phase 3/4 clinical trials of EGFR or ErbB family TKIs as first-line therapy in *EGFR* mutation-positive or clinically selected advanced NSCLC

Trial	Estimated enrollment	Key eligibility	Treatment arm(s)	Primary outcome	Status^1^
Gefitinib					
Phase 4 (Europe)—NCT01203917	1060	Caucasian race *EGFR* mutation-positive disease	Gefitinib	RR	Active, not recruiting
Erlotinib					
Phase 3—NCT01667562	30	*EGFR* mutation-positive disease	Erlotinib	PFS	Active, not recruiting
Afatinib					
Phase 3—NCT01121393 (LUX-Lung 6)	364	Adenocarcinoma *EGFR* mutation-positive disease	Afatinib vs. cisplatin/gemcitabine	PFS	Active, not recruiting
Phase 3—NCT00949650 (LUX-Lung 3)	345	Adenocarcinoma *EGFR* mutation-positive disease	Afatinib vs. cisplatin/pemetrexed	PFS	Active, not recruiting
Dacomitinib					
Phase 3—NCT01774721 (ARCHER 1050)	440	Known histology *EGFR* mutation-positive disease, specifically exon 19 deletion or L858R mutation in exon 21 (may occur with an exon 20 T790M mutation)	Dacomitinib vs. gefitinib	PFS	Active, not recruiting

EGFR, epidermal growth factor receptor; TKIs, tyrosine kinase inhibitors; NSCLC, non-small-cell lung cancer; RR, response rate; PFS, progression-free survival.

1 Per the U.S. National Institutes of Health ClinicalTrials.gov database, accessed July 2015.

#### Irreversible ErbB family TKIs (afatinib and dacomitinib)

Afatinib is an irreversible ErbB family inhibitor of EGFR/Erb1, ErbB2/HER2, and ErbB4/HER4 29,30. It has also been shown to inhibit phosphorylation of ErB3/HER3 in vitro 31. In the LUX-Lung 2 phase 2 trial of first-line or second-line afatinib in *EGFR* mutation-positive advanced or recurrent NSCLC (*N* = 129), median PFS was 10.1 months, median OS was 24.8 months, and the independent confirmed RR was 61% 32. In subgroups based on specific mutation type, the RR was 66% among 106 patients with common mutations (exon deletion 19 or exon 21 L858R) and 39% among 23 patients with other mutations. Results are also available from phase 3 trials (LUX-Lung 3 and LUX-Lung 6) of afatinib in patients with *EGFR* mutation-positive lung adenocarcinoma (Table1) 33,34. Data from LUX-Lung 6, which evaluated afatinib versus cisplatin plus gemcitabine in Asian patients, showed significantly prolonged PFS with afatinib versus gemcitabine/cisplatin (11.0 vs. 5.6 months, respectively; *P *<* *0.0001) and a significantly higher RR (66.9% vs. 23.0%; *P *<* *0.0001) by independent review 33. The global LUX-Lung 3 study evaluated afatinib versus pemetrexed/cisplatin and also showed significantly prolonged PFS with afatinib both overall (11.1 vs. 6.9 months; *P *=* *0.001) and in patients with common (exon 19 deletions or L858R) *EGFR* mutations (13.6 vs. 6.9 months; *P *<* *0.0001) by independent review; results also showed a significantly higher RR (56% vs. 23%; *P *=* *0.001) and delayed worsening of lung cancer-related symptoms with afatinib 34–36. Of note, randomization in both the LUX-Lung 6 and LUX-Lung 3 trials was stratified by type of *EGFR* mutation (L858R, Del19, or other); efficacy analyses by *EGFR* mutation type were prespecified 33,35. Analysis of OS by *EGFR* mutation type in LUX-Lung 6 and LUX-Lung 3 revealed an OS benefit in afatinib-treated patients with Del19 mutations (Table2) 34. In LUX-Lung 6, the median OS in patients with Del19 *EGFR* mutation treated with afatinib versus chemotherapy was 31.4 versus 18.4 months (*P *=* *0.0229). However, no significant differences in OS were observed between treatment groups among patients with L858R mutation. Similarly, in LUX-Lung 3, afatinib-treated patients in the Del19 subgroup demonstrated prolonged OS compared with chemotherapy-treated patients (33.3 vs. 21.1 months; *P *=* *0.0015), but no significant OS differences were observed between the two treatment arms in patients with L858R mutation.

Dacomitinib (Pfizer, New London, CT) is an irreversible pan-HER inhibitor of EGFR/ErbB1, ErbB2/HER2, and ErbB4/HER4 37. A phase 3 trial, ARCHER 1050 (NCT01774721), will evaluate dacomitinib versus gefitinib in the first-line treatment of *EGFR* mutation-positive advanced NSCLC (Table3). An ongoing phase 3 trial of dacomitinib in advanced NSCLC unresponsive to standard therapy, including one to three lines of chemotherapy and an EGFR TKI (BR26; NCT01000025), is not focused on molecularly selected patients; however, its secondary endpoints include OS in *EGFR* mutation-positive or wild-type *KRAS* subsets. Preliminary results were recently presented and showed that dacomitinib did not improve OS versus placebo (6.8 vs. 6.3 months; *P *=* *0.99), but did significantly improve PFS (2.7 vs. 1.4 months; *P *<* *0.0001) and RR (7% vs. 1%; *P *=* *0.001) 38. Effect of dacomitinib on OS was similar regardless of *EGFR* mutation status; however, OS results appeared to differ by *KRAS* mutation status, with dacomitinib improving OS in patients with *KRAS* wild-type tumors (7.0 vs. 5.2 months; hazard ratio [HR], 0.79; 95% confidence interval [CI], 0.61–1.03), but worsening OS in patients with *KRAS* mutation-positive NSCLC (5.8 vs. 8.3 months; HR, 2.1; 95% CI, 1.05–4.22; interaction *P *=* *0.08). In a randomized phase 2 trial of dacomitinib versus erlotinib after one or two lines of chemotherapy for advanced NSCLC, PFS was prolonged with dacomitinib in the overall population (2.86 vs. 1.91 months for erlotinib; *P *=* *0.012) and most clinically or molecularly defined subgroups, including patients with *EGFR* mutation-positive disease (3.71 vs. 1.91 months; *P *=* *0.006) 39. Preliminary results are available from a phase 2 trial of first-line dacomitinib in 74 patients with adenocarcinoma who were either nonsmokers/former light smokers or had documented *EGFR* mutations; median PFS was 9.30 months in all patients, but had not been reached in the 27-patient subset with confirmed *EGFR* mutations (all of whom had experienced tumor shrinkage) 40. In the *EGFR* mutation-positive subgroup, 4-, 6-, and 9-month PFS rates were 95.7%, 84.7%, and 84.7%, respectively (higher than those in the overall population, which were 73.3%, 67.0%, and 57.1%, respectively). In an updated analysis of 47 patients with *EGFR* mutations involving exons 19 or 21, the partial RR was 74% and 1-year and median PFS were 77% and 17 months, respectively 41. Preliminary results from an ongoing phase 3 trial (ARCHER 1009) in unselected patients with advanced NSCLC suggest similar PFS with dacomitinib versus erlotinib as second- or third-line therapy in the coprimary populations, all patients (2.6 vs. 2.6 months; *P *=* *0.229) and *KRAS* wild-type patients (2.6 vs. 2.6 months; *P *=* *0.587); OS and outcomes for patients with *EGFR* mutation are not mature 42.

## Compensatory ErbB Family Signaling

Just as there are several sensitizing *EGFR* mutations that predict response to EGFR TKIs, other mutations have been linked to acquired resistance. The T790M mutation in exon 20 was the first identified mechanism of acquired resistance to EGFR TKIs and is thought to influence receptor affinity toward ATP and occur in at least 50% of cases of acquired resistance to EGFR-TKI therapy 43. Interestingly, patients with T790M mutations have been shown to have a more favorable disease course in the postprogression period relative to patients with acquired resistance without T790M mutation 44. Few secondary mutations other than T790M have been identified to date and include D761Y, L747S, and T854A; these non-T790M mutations are thought to occur in <5% of *EGFR*-mutated TKI-resistant patients 43. To specifically evaluate mechanisms of acquired resistance to EGFR-TKI therapy for NSCLC, investigators at the Memorial Sloan Kettering Cancer Center implemented a prospective registry (NCT00579683) to compare *EGFR* gene sequence at relapse versus prior to EGFR-TKI therapy. Secondary outcomes include identification of novel *EGFR* mutations and resistance mechanisms and a more precise characterization of the frequency and clinical implications of T790M mutations.

**Figure 2:**
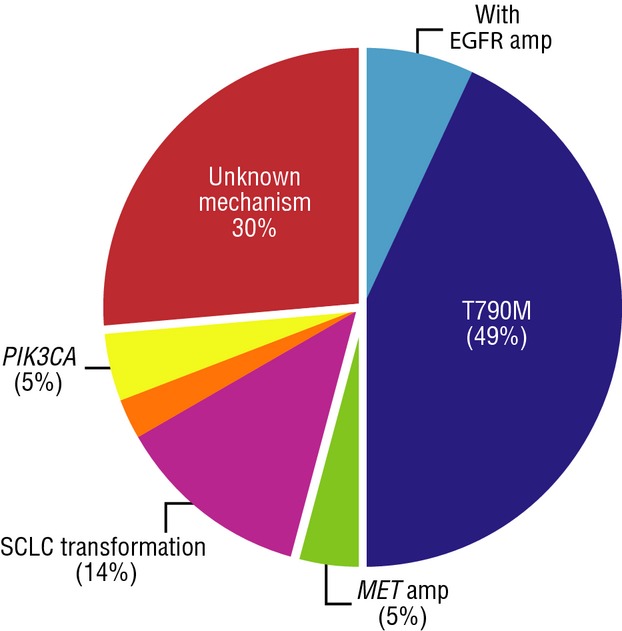
Frequency of observed drug resistance mechanisms in 37 patients with NSCLC biopsied at the time of acquired resistance. Note that orange wedge represents one patient who had both SCLC transformation and acquisition of a *PIK3CA* mutation. EGFR, epidermal growth factor receptor; MET, met proto-oncogene; *PIK3CA*, phosphatidylinositol-4,5-bisphosphate 3-kinase, catalytic subunit alpha; NSCLC, non-small-cell lung cancer; SCLC, small-cell lung cancer. From Sequist et al. 53. Adapted with permission from AAA.

A number of signaling pathways share downstream targets with EGFR and have been implicated in resistance to EGFR TKIs (Fig.1) 43, including hepatocyte growth factor receptor (MET) 45, AXL receptor TK 46, and nuclear factor kappa-light-chain-enhancer of activated B cells (NF-*κ*B) 47,48. Other resistance mechanisms include *ERBB2/HER2* amplification 49, epithelial-to-mesenchymal transition (EMT) 50, and BIM polymorphism 51. In addition, PI3K/Akt/mTOR (downstream mediator of EGFR signaling) may function as a compensatory EGFR signaling pathway; mutations in the main catalytic subunit of PI3K (*PIK3CA*) have been associated with primary and acquired EGFR-TKI resistance, coexisting with *EGFR* mutations in some cases 52,53. Systematic genetic and histologic analyses of tumor biopsy specimens from 37 patients with drug-resistant *EGFR* mutation-positive NSCLC were performed to determine mechanisms of acquired resistance and found that all tumors retained their pretreatment activating *EGFR* mutations and many acquired other resistance mechanisms, including T790M or *MET* gene amplification (Fig.2) 53. The authors also reported histology transformation in tumors with acquired resistance; five patients with lung adenocarcinoma before EGFR-TKI treatment were found to have small-cell lung cancer (SCLC) in drug-resistant tumor biopsies, while retaining the original *EGFR* mutation 53. This transition from NSCLC to SCLC appears to be specific to EGFR-TKI resistance and supports the importance of repeat biopsies at the time of resistance.

Agents that target each of these various resistance pathways are in clinical development, with several evaluated in completed or ongoing phase 3 trials in molecularly selected NSCLC populations.

As *MET* oncogene amplification has been implicated in both primary and acquired resistance to EGFR inhibition 54, MET inhibitors are being studied in combination with erlotinib for molecularly selected, advanced NSCLC. The combination of onartuzumab (MetMAb; Genentech, South San Francisco, CA), a monoclonal antibody (mAb) targeting the MET receptor 55, plus erlotinib is being evaluated in a phase 3 trial in previously treated, MET-expressing (by IHC), advanced NSCLC (NCT01456325). Based on preliminary data suggesting that the addition of onartuzumab to erlotinib did not improve OS (6.8 vs. 9.1 months; *P *=* *0.068), PFS (2.7 vs. 2.6 months; *P *=* *0.92), or RR (8.4% vs. 9.6%; *P *=* *0.63) versus erlotinib/placebo, an independent data review committee recommended stopping the trial for futility; however, exploratory analyses by molecular subgroups are pending 56. In a placebo-controlled phase 2 trial of onartuzumab plus erlotinib in 137 unselected, previously treated patients with advanced NSCLC, onartuzumab recipients with MET-expressing (by IHC) tumors had significantly prolonged PFS (*P *=* *0.04) and OS (*P *=* *0.002) 57. Conversely, onartuzumab was associated with shortened PFS in the MET expression-negative setting (*P *=* *0.05). Tivantinib (ARQ 197; ArQule, Inc., Woburn, MA), a MET TKI 58, is being evaluated in combination with erlotinib in a phase 3 trial in Asian patients with wild-type *EGFR* advanced NSCLC (ATTENTION; NCT01377376). Preliminary data from the trial suggest some benefit from adding tivantinib to erlotinib in this patient population (OS: 12.9 vs. 11.2 months with placebo/erlotinib, *P *=* *0.427; PFS: 2.9 vs. 2.0 months; *P *=* *0.019); however, the trial lacked statistical power due to premature termination for toxicity concern (imbalance in interstitial lung disease between groups) 59. In a placebo-controlled phase 2 trial of tivantinib plus erlotinib in 167 unselected, previously treated patients with advanced NSCLC, tivantinib/erlotinib recipients with wild-type *EGFR* had numerically improved PFS (*P *=* *0.25) and OS (*P *=* *0.25) 60. Tivantinib/erlotinib-treated patients with *KRAS* mutations had significantly improved PFS (*P *<* *0.01) and numerically improved OS (*P *=* *0.17).

Another approach to attenuating acquired resistance to EGFR TKIs is to prevent the associated epigenetic changes and EMT that have been observed during EGFR-TKI therapy 61. The histone deacetylase inhibitor entinostat, which has these capabilities, was evaluated in a placebo-controlled phase 2 trial with erlotinib in chemotherapy-pretreated, molecularly unselected, advanced NSCLC 61. The combination of erlotinib plus entinostat did not improve 4-month PFS (18% vs. 20% with erlotinib/placebo; *P *=* *0.7) or other efficacy outcomes. However, a planned biomarker analysis found that the 26-patient subset with high baseline expression of E-cadherin had significantly longer OS with entinostat/erlotinib versus erlotinib alone (9.4 vs. 5.4 months; *P *=* *0.03) and numerically prolonged PFS (3.7 vs. 1.9 months; *P *=* *0.19), supporting further study of the combination in patients with high E-cadherin expression.

## Conclusions and Future Directions

Personalized therapy is now a clinical reality in NSCLC—an era that began with the reversible EGFR TKIs gefitinib and erlotinib for *EGFR* mutation-positive disease. Interim data for several phase 3 trials of gefitinib or erlotinib monotherapy versus doublet chemotherapy were favorable enough to warrant early study closures, with subsequent treatment crossover as a confounding factor in the analysis of OS. In contrast, data show less favorable outcomes with EGFR TKIs versus conventional platinum-based chemotherapy in patients with *EGFR* wild-type NSCLC and thus EGFR TKIs cannot be recommended in the first-line metastatic setting without evidence of a sensitizing *EGFR* mutation 16. Results are awaited from ongoing phase 3 trials of investigational treatments and EGFR-TKI-containing combination regimens in molecularly selected NSCLC. We recommend that *EGFR* mutation testing be done both at the time of diagnosis for patients who are suitable for therapy and also considered at the time of recurrence or progression in an effort to determine the mechanism(s) of resistance and to more effectively direct future therapies 16. Recent data suggest clinical benefits with the continuation of EGFR TKIs beyond progression in patients developing acquired resistance 1,16,62. In fact, discontinuation of EGFR-TKI therapy in patients who were once sensitive to EGFR inhibition may lead to more rapid cancer progression 63,64. In terms of treatment options when genomic data are unavailable, erlotinib has been shown to significantly prolong PFS and OS compared with placebo irrespective of *EGFR* mutation status both as switch maintenance therapy following conventional chemotherapy and in patients with NSCLC after failure of first- or second-line chemotherapy 14,65. Similarly, continuing treatment with afatinib in patients with metastatic NSCLC who had progressed following treatment with reversible EGFR TKIs and afatinib revealed that afatinib treatment beyond progression significantly improves PFS and objective response rate versus chemotherapy alone 62.

To date, clinical investigations of EGFR-directed therapy for *EGFR* mutation-positive NSCLC have focused on the use of TKI monotherapy for advanced disease. Molecularly focused evaluations of other types of therapies, such as anti-EGFR therapeutic vaccines 66 or TKI/mAb combinations that more broadly target the ErbB family (e.g., erlotinib plus the HER2-targeted mAb pertuzumab 67, and afatinib plus the EGFR-targeted mAb cetuximab 68), may be worthwhile. Outstanding questions include whether EGFR TKIs confer clinical benefit when used in less advanced disease, which will be addressed by ongoing and recently completed phase 3 trials of gefitinib or erlotinib versus combination chemotherapy in the adjuvant or neoadjuvant setting for patients with *EGFR* mutation-positive NSCLC (NCT01405079 [ADJUVANT trial of gefitinib vs. vinorelbine/platinum stage II–IIIA(N1–N2) disease]; NCT01407822 [EMERGING trial of erlotinib vs. gemcitabine/cisplatin as neoadjuvant therapy for stage IIIA(N2) disease], and NCT00373425 [RADIANT trial of erlotinib vs. placebo added to adjuvant chemotherapy for stage IB–IIIA disease]). Preliminary results from the RADIANT trial were recently presented and suggest that adjuvant erlotinib may prolong disease-free survival (DFS) in patients with *EGFR* mutation-positive NSCLC, but adjuvant erlotinib did not significantly improve DFS over placebo in the overall patient population 69,70. There is also a need for pooled testing of targets with limited quantities of tissue for testing and the centralization of data repositories for this information.
